# A comprehensive repertoire of tRNA-derived fragments in prostate cancer

**DOI:** 10.18632/oncotarget.8293

**Published:** 2016-03-23

**Authors:** Michael Olvedy, Mauro Scaravilli, Youri Hoogstrate, Tapio Visakorpi, Guido Jenster, Elena S. Martens

**Affiliations:** ^1^ Department of Urology, Erasmus MC, Rotterdam, The Netherlands; ^2^ Institute of Biosciences and Medical Technology-BioMediTech, University of Tampere, Tampere, Finland; ^3^ Fimlab Laboratories, Tampere University Hospital, Tampere, Finland; ^4^ Current address: VIB Center for the Biology of Disease, KU Leuven, Leuven, Belgium; ^5^ Current address: Center for Human Genetics, KULeuven, Leuven, Belgium

**Keywords:** tRNA-derived fragments (tRFs), prostate cancer (PCa), RNA-sequencing, non-coding RNA, biomarker

## Abstract

Prostate cancer (PCa) is the most common cancer among men in developed countries. Although its genetic background is thoroughly investigated, rather little is known about the role of small non-coding RNAs (sncRNA) in this disease. tRNA-derived fragments (tRFs) represent a new class of sncRNAs, which are present in a broad range of species and have been reported to play a role in several cellular processes. Here, we analyzed the expression of tRFs in fresh frozen patient samples derived from normal adjacent prostate and different stages of PCa by RNA-sequencing. We identified 598 unique tRFs, many of which are deregulated in cancer samples when compared to normal adjacent tissue. Most of the identified tRFs are derived from the 5’- and 3’-ends of mature cytosolic tRNAs, but we also found tRFs produced from other parts of tRNAs, including pre-tRNA trailers and leaders, as well as tRFs from mitochondrial tRNAs. The 5’-derived tRFs comprise the most abundant class of tRFs in general and represent the major class among upregulated tRFs. The 3’-derived tRFs types are dominant among downregulated tRFs in PCa. We validated the expression of three tRFs using qPCR. The ratio of tRFs derived from tRNA^LysCTT^ and tRNA^PheGAA^ emerged as a good indicator of progression-free survival and a candidate prognostic marker. This study provides a systematic catalogue of tRFs and their dysregulation in PCa and can serve as the basis for further research on the biomarker potential and functional roles of tRFs in this disease.

## INTRODUCTION

Prostate cancer (PCa) is the second most common cancer in men worldwide [1]. The treatment of PCa is hampered by the lack of reliable markers for disease outcome prediction leading to incorrect patient stratification, overtreatment and consequent side effects from prostatectomy and radiation therapy [2]. A better understanding of the molecular mechanisms behind the onset and progression of PCa is needed in order to discover better markers and develop new therapeutic strategies. The role of small non-coding RNAs (sncRNAs) other than microRNAs (miRNAs) in PCa is poorly understood. The rapid progress and popularity of high throughput sequencing led to the discovery of a novel class of sncRNAs derived from tRNAs and named tRNA-derived fragments (tRFs) [3–5]. tRFs are present across all domains of life [6–8]. While initially considered random products of tRNA turnover, their abundance and ubiquitous expression suggest that tRFs are actual biological entities [6, 7].

tRFs can be generated by endonucleases such as ribonuclease T2 (Rny1p) in yeast and angiogenin or dicer 1 in human. Based on size, they are divided into two groups. The first group consists of tRFs with a size of 30 to 35 nt, which are generally referred to as tRNA halves or stress-induced tRFs. tRNA halves are produced by endonucleolytic cleavage at the anticodon loop of the full-length tRNA. The second group consists of tRFs with a size of about 20 nt and can be further divided into 5’- and 3’-derived tRFs, originating from the 5′- and 3′-parts of mature tRNAs, respectively [4, 9, 10]. The small RNAs derived from the 5’-leader and 3’-trailer sequences of the precursor tRNAs (pre-tRNAs) are also classified as tRFs [5, 11, 12].

Expression of tRFs is detected in different cancer cell lines, including the PCa cell lines LNCaP and C4-2 [4, 5, 13–15]. In a previous study, we reported the discovery and differential expression of tRFs in clinical samples of PCa [16]. This suggests that tRFs might play an important role in the pathogenesis of cancer. The mechanism behind the function of tRFs appears to be diverse. Several reports demonstrate that tRF levels are elevated by cellular stress conditions and particularly under oxidative stress such as hypoxia [10, 13, 15, 17]. tRFs are also involved in post-transcriptional regulation of gene expression via direct inhibition of protein synthesis by displacing the eIF4G translation initiation factor from mRNA [18–20]. Moreover, a 3′-derived tRF identified in B-cell lymphoma cells possesses the functional characteristics of a guide RNA that suppresses proliferation and modulates response to DNA damage in a miRNA-fashion [21]. It has also been shown that tRFs can compete for the binding sites of the RNA-binding protein YBX1, which is involved in the stabilization of oncogenic transcripts suppressing cell growth and invasion [15]. In this way, tRFs antagonize the activity of YBX1 and act as tumor suppressors. Taken together, these findings strongly suggest a functional role of tRFs in tumorigenesis.

Very recently, it was proposed that although tRFs are defined biological entities, their composition and abundance in the transcriptome is dependent on gender, tissue, disease and even disease subtype [22]. This suggests that tRFs can be explored as novel sensitive biomarkers of disease. Yet, studies providing systematic insight into the composition and expression of the tRF transcriptome throughout various disease stages are still missing. Here, we analyze tRF expression in an extended cohort of clinical samples representing progressing stages of PCa. We construct a database of tRFs expressed across PCa samples and identify the most differentially expressed tRFs. Finally, we perform a qPCR quantification in two cohorts of clinical samples to validate the differential expression of selected tRFs.

## RESULTS

### Inventory of tRFs expressed in PCa

In order to obtain a global overview of the tRF repertoire in PCa, we analyzed tRFs across normal adjacent prostate (NAP), benign-prostate hyperplasia (BPH), PCa from radical prostatectomies, trans-urethral resected tissue from castration resistant PCa (TURP_PCa), and lymph node metastasis (LN_PCa) using next-generation RNA sequencing (Table 1).

**Table 1:** Clinical parameters of the samples used for the RNA-sequencing

Group name	Patient samples	TMPRSS2_ERG fusion	ETV1 aberrations	Cancer cells (%)	Gleason score	Preoperative PSA Mean (Min.- Max.)	Status after radical prostatectomy*
***NAP***	4	0 (0%)	0	0	N/A	5.8 (2.5 - 11)	N/A
***BPH***	4	N/A	N/A	0	N/A	N/A	N/A
***PCa6_cur***	4	4 (100%)	0	70-90	3+3	3.8 (2.0 - 7.2)	Cured
***PCa6_nofusion***	4	0 (0%)	0	70-90	3+3	10.8 (0.5 - 23.7)	Recurrent
***PCa6_TERG***	4	4 (100%)	0	80-90	3+3	7.3 (2.0 - 10.5)	Recurrent
***PCa6_recur***	4	4 (100%)	0	80-90	3+3	11.3 (6.0 - 64.3)	Recurrent
***PCa7_recur***	4	2 (50%)	1 (fusion)	80-100	4+3	25.2 (6.5 - 64.3)	Recurrent
***PCa8_recur***	3	1 (25%)	1 (overexpressed)	90-100	4+4 (5)	32.4 (31.9 - 32.9)	Recurrent
***TURP_PCa (castration resistant)***	4	1 (25%)	1 (fusion)	90-100	(3+4) to (5+4)	Unknown	Recurrent
***LN_PCa***	4	3 (75%)	1 (fusion)	100	4+4(5)†	154.6 (80.2 - 252.5)	N/A

* Patients were considered cured if there was no biochemical relapse or detection of metastasis after radical prostatectomy

† Gleason score of the primary tumor

**Group abbreviations:** NAP, normal adjacent prostate; BPH, benign prostatic hyperplasia; PCa, organ-confined prostate cancer; cur/recur, cured/recurrent after radical prostatectomy; PCa6_nofusion, PCa Gleason score 3+3 with no TMPRSS2- ERG fusion or ETV abnormalities; PCa6_TERG, PCa Gleason score 3+3 with TMPRSS2-ERG fusion; TURP_PCa, trans-urethral resection of the prostate, castration resistant PCa; LN_PCa, lymph node metastasis.

**Figure 1:**
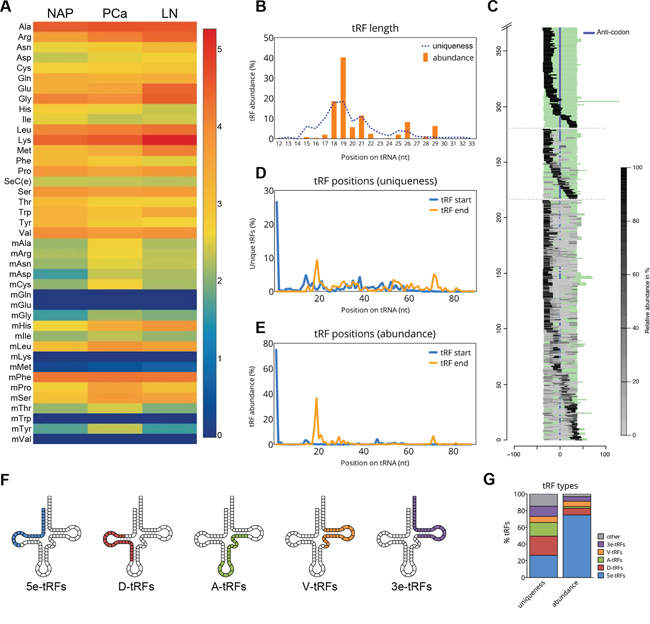
tRF types in prostate cancer **A.** Heatmap showing the total read counts mapped to individual tRNA isotypes in three study groups: NAP, normal adjacent prostate; PCa, prostate cancer group (consisting of 6 different sample pools, the average value is shown); LN_PCa, lymph node metastasis. The color and its corresponding value in log_10_ scale are depicted on the right. **B.** tRF length as based on the read abundance (orange bars) and uniqueness (dotted line). **C.** Graph depicting the locations of mapped tRFs on the sequences of mature tRNAs. Full-length tRNA sequences are aligned to the middle using the anticodon position. tRFs mapped to these tRNAs are depicted as grey bars which relative abundance per particular tRNA is reflected by the color intensity (light grey, low abundance; black, high abundance). tRNAs with only one mapped tRF are clustered at the top, tRNAs with two mapped tRFs in the middle and tRNAs with multiple mapped tRFs are at the bottom. **D-E.** Start (blue line) and end (orange line) positions of tRFs on the mature tRNA sequence. Relative abundance of each tRF start and end based on the uniqueness (D) or abundance (E) is shown. **F.** An illustration of various tRF types and their approximate location on the secondary structure of tRNA. **G.** Relative proportions of each tRF types in our dataset as based on the uniqueness (% of unique independent reads) or abundance (% of total number of reads).

All 21 cytosolic tRNA isotypes (including selenocystein tRNAs) were found to produce tRFs in variable amounts (Figure 1A). tRNA^Ala^ and tRNA^Lys^ showed the highest numbers of mapped tRFs, while the least tRFs were produced from tRNA^Ile^ and tRNA^Asp^. The raw sum of tRFs weakly correlated with the number of tRNA genes per isotype or anticodon, as well as with the percentage of codon usage (Supplementary Figure 1; codon usage from http://gtrnadb.ucsc.edu/Hsapi19/Hsapi19-summary-codon.html). tRFs derived from 15 out of 20 mitochondrial tRNAs (mtRNAs) were also detected (Figure 1A). We could not detect tRFs corresponding to the mitochondrial tRNA isotypes mtRNA^Gln^, mtRNA^Glu^, mtRNA^Lys^, mtRNA^Trp^ and mtRNA^Val^. With the exception of mtRNA^Phe^, most mtRNA isotypes had a lower number of mapped tRFs, compared to cytosolic tRNAs. The read count of mtRNA^Phe^ in the NAP group was 83-fold higher than the average of all other mtRNA read counts.

In order to quantify the expression of tRFs, we assembled a PCa tRF-database using the fragment detection algorithm FlaiMapper [23]. The read-coverage of mature cytoplasmic tRNAs across all groups was analyzed using CLC-Bio Genomics Workbench. Initially, 1175 tRFs were identified and mapped to 386 unique cytosolic tRNAs [24]. However, since tRNA sequences are highly conserved within tRNA isotypes, some tRFs were mapped to more than one unique tRNA (Supplementary Figure 2) and the total read-count in the initial mapping was equally divided across them. Upon further examination, we noticed that this causes underrepresentation of sequence counts for tRFs that had identical sequence but could be mapped to multiple tRNA isotypes. Therefore, tRFs with identical sequences were merged into single entries, even if they could be derived from different tRNAs, and their corresponding reads were summed. After this correction, a total of 598 unique tRFs were identified (Supplementary Table 1). Multiple fragmentation patterns, in combination with low read-count, caused low reliability in the automated prediction of tRFs derived from mtRNAs (Supplementary Figure 3). Therefore, these tRFs were omitted from further analysis.

Based on their size, tRNA-derived fragments can be generally separated into two major categories: tRNA halves, with a size of 30-35 nt and small tRNA fragments (tRFs), with a size of approximately 20 nt. In our dataset, small tRFs were predominant and their sizes ranged from 15 to 23 nt (Figure 1B). The most abundant tRFs, however, were between 18 to 21 nt, while 40% of tRFs were 19 nt long (Figure 1B). A group of longer tRFs, with sizes between 25 and 29 nt, was also identified.

In addition to tRFs derived from mature tRNAs, we were also able to detect fragments corresponding to the 5’-pre-tRNA leader (5’U-tRFs) and 3’-pre-tRNA trailer (3’U-tRFs) sequences of various tRNAs (Supplementary Table 2). The length of 5’U-tRFs and 3’U-tRFs varied between 15 and 25 nt. Most 5’U-tRFs were 17 nt long and most 3’U-tRFs were 18 nt long (Supplementary Figure 4A). Interestingly, more than 54% of 3’U-tRFs and 30% of 5’U-tRFs were derived from sequences right next to or 1 nt away from the mature tRNA sequence (Supplementary Figure 4B-4C), suggesting that they are produced during the normal processing of pre-tRNA. Both 5’U-tRFs and 3’U-tRFs showed overall low expression values (data not shown), with the exception of tRF-1001/cand45. This fragment was previously detected in PCa cell lines, as well as in human colon carcinoma and human embryonic kidney cells [5, 11]. In our libraries tRF-1001/cand45 showed read counts from 40 000 in the NAP and PCa (average) groups to 110 000 in the LN_PCa group.

### tRFs derived from the 5’-end are dominant in PCa

The majority of tRFs identified in our samples originate from the 5′- and the 3′-end of tRNAs (Figure 1C). This is in concordance with previous studies collectively reporting on the existence of short tRFs derived from the 5′- and the 3′-end of mature tRNAs [5, 25, 26]. To analyze the relative abundance of each tRF class in our dataset we examined the start and end positions of all unique tRFs on their precursor tRNAs. All the fragments with a 3’-end nucleotide at position ≤40 on the mature tRNA sequence were considered as 5′-derived, whereas all fragments with the first 5’-nucleotide at position ≥30 on the mature tRNA sequence were considered as 3′-derived. Based on fragment uniqueness, we found comparable rates of tRF types, *i.e.* 51.7% corresponded to 5′-derived tRFs and 44.2% to 3′-derived tRFs. Nevertheless, when relative fragment abundance was taken into account a strong bias towards the 5′-derived (84.7%) *vs.* the 3’-derived tRFs was observed.

To get a more precise overview of the localization of tRFs, we also analyzed their start- and end- position frequencies. Interestingly, more than 26% of all unique tRFs, which in the terms of abundance account for over 80% of all tRFs, were found to start at position 1 on the mature tRNA sequence (Figure 1D–1E). Most of these tRFs have the end at position 19 on the mature tRNA. Based on the peaks generated by the start positions of all unique fragments (Figure 1D), we observed that the tRF pool constitutes of several distinct classes (note the peak appearing before 20 nt, another at around 40 nt and another before 60 nt of the mature tRNA). While categorizing tRFs into 5′- or 3′-derived tRFs is very common, we found that at least 5 different classes are present across our samples. Therefore, we classified tRFs into (i) 5e-tRFs with a start position in the first nucleotide of the 5’-end of the tRNA (“e” stands for “end”); (ii) D-tRFs with a start position between nucleotides 12-23 and overlapping the D-loop of the precursor-tRNA; (iii) A-tRFs starting between nucleotides 31-39 and overlapping with the anticodon loop; (iv) V-tRFs with a start between nucleotides 45-49 and overlapping the variable loop; and finally, (v) 3e-tRFs starting between nucleotides 50-60 and overlapping the T loop (Figure 1F). While 5e-tRFs represent the most abundant class of tRFs (approximately 75%), other classes of tRFs appear to have very similar expression (<10% abundance) compared to each other (Figure 1G). Interestingly, similar tRF types have been detected in the lower eukaryote *Tetrahymena thermophile*, suggesting the existence of an evolutionary conserved tRNA processing mechanisms [27]. Moreover, the position of these peaks was found to overlap with all tRNA loops, indicating that endonucleolytic cleavage occurs in the single-stranded loop regions of tRNAs.

### Several tRFs are deregulated in PCa

To investigate whether tRF production is dysregulated in PCa, we compared the expression levels of tRFs in normal tissue and in samples from different clinical stages representing progressing disease (Table 1). While expression levels of other types of sncRNAs correlated well between the two libraries representing non-malignant tissue, *i.e.* NAP and BPH (Pearson r=0.89, P-value <0.0001; median fold-change −0.002), tRFs showed lower correlation and a very high one-directional deviation towards increased expression in the BPH library (Pearson r=0.81; P-value <0.0001; median fold-change 0.758; Supplementary Figure 5). These results indicate that tRFs, as opposed to other sncRNAs, might be differentially expressed in benign prostate hyperplasia. This difference can be explained by the different anatomical origin of the BPH and NAP/PCa samples. While BPH occurs exclusively in the transition zone of the prostate, prostate tumors are predominantly localized in the peripheral zone. Both zones are characterized by distinct expression profiles indicating differential regulation of a large number of genes [28]. For this reason, BPH was excluded as a control sample from further analyses. Only NAP tissue, confirmed to contain 0% tumor cells by two independent pathologists, was used as a control sample. It should be acknowledged, that adjacent tissue could be altered in gene/ncRNA expression due to tumor-stroma paracrine influences [29]. Nevertheless, as much as stroma/epithelium interactions are unavoidable in experiments were samples are derived by macro-dissection, much care has been taken to minimize the influence of the stromal compartment on the expression profiles of small RNAs by selecting tissue sections of at least 70% epithelial cells [24].

**Figure 2:**
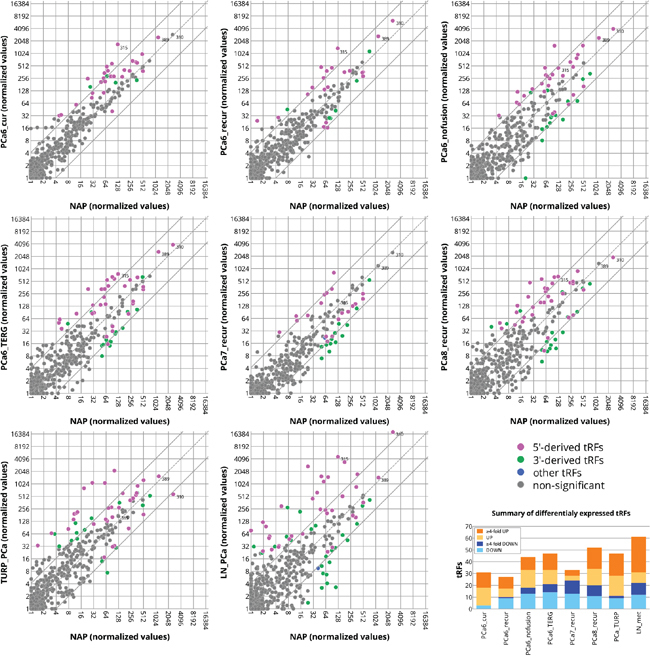
Differentially expressed tRFs in prostate cancer Plotted are normalized read-count values of each tRF in the normal adjacent prostate versus various stages of prostate cancer. The baseline value for tRFs that are not expressed is 1. Full lines represent 4-fold change borderlines. Colored points represent significantly changed tRFs (Kal's Z-test on proportions, Bonferroni corrected p-values, p < 0.05) labeled as 5’-derived (magenta) and 3’-derived (green) tRFs. tRFs with the 3’-nucleotide at a position ≤40 on the precursor tRNA sequence are considered as 5′-derived. tRFs with the start nucleotide at a position ≥30 on the precursor tRNA sequence are considered as 3′-derived. tRFs that do not fall into either of these two categories are shown in blue. Positions of tRF-310, tRF-315, and tRF-389 are indicated as an example of three differentially expressed tRFs. The graph at the bottom right corner summarizes the total number of differentially expressed tRFs per group. The amount of tRFs with ≥4-fold differential expression are indicated in dark orange (upregulated) or dark blue (downregulated) color.

We found several tRFs to be significantly differentially expressed in PCa when compared to NAP (Kal's Z-test with Bonferroni correction, p-value < 0.05) (Figure 2 and Supplementary Table 3-4). The number of differentially expressed tRFs varied slightly between the stages of PCa, with a minimum of 27 differentially expressed tRFs in PCa6_recur group and a maximum of 61 differentially expressed tRFs in the LN_PCa group (Figure 2, Supplementary Table 4). We identified 12 tRFs to be commonly differentially expressed between recurrent PCa groups with Gleason grade 6, 7, or 8 (Supplementary Table 5). Of these, 5 were upregulated, 6 downregulated and 1 was downregulated in PCa6 group but upregulated in PCa7 and PCa8 groups (Supplementary Table 5). This result indicates that a small subset of differentially expressed tRFs can be found across increasing grades of PCa.

In summary, we found 110 differentially expressed tRFs across our dataset, out of which 72 were upregulated, 24 downregulated and 13 that were upregulated in one but downregulated in other group.

### tRFs deregulated in PCa belong to distinct classes

**Figure 3:**
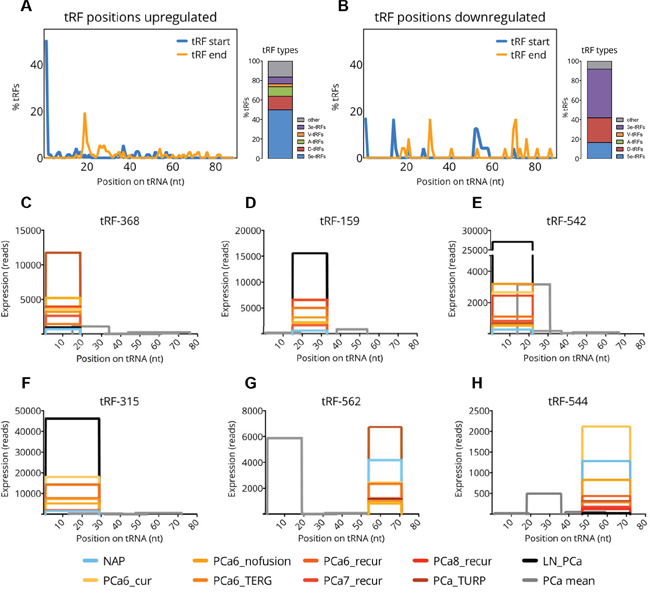
Frequency of tRF types among differentially expressed tRFs **A-B.** Start (blue line) and end (orange line) positions of tRFs on the mature tRNA sequence and the proportions of each tRF type for 72 upregulated (A) and 24 downregulated (B) tRFs. **C-H.** Graphs showing the exact positions of 6 selected tRFs (shown in color) and other tRFs (shown in grey) on their tRNA precursors. The x-axis represents the position on the precursor sequence in nucleotides. The y-axis represents summed read-counts per sample group. The expression level per group is indicated by different color (see the legend at the bottom). Expression levels of other tRFs are shown as means across PCa groups.

It has been proposed that 5′- but not 3′-derived tRFs, play a role in stress granule assembly or inhibition of protein synthesis *in vitro* [19, 30]. On the other hand, some 3′-derived tRFs are able to repress their mRNA targets in a miRNA-like fashion and may exert tumor suppressive functions [21, 31]. Interestingly, our results indicate that the deregulation of 5’-derived tRFs differs from that of 3’-derived tRFs (Figure 2). In order to study which tRF types are present among the downregulated and upregulated tRFs in PCa we compared the percentage of different tRF types among our groups of upregulated and downregulated tRFs (Figure 3A–3B). We noticed major differences in the abundance of tRF types in both lists. Most of the upregulated tRFs were 5e-tRFs (50%) and most downregulated were 3e-tRFs (50%). We selected tRFs originating from 6 different tRNAs for further analysis and qPCR validation. All of them were commonly differentially regulated in recurrent PCa groups with Gleason grade 6, 7, or 8 (Supplementary Table 5). Out of these, 4 tRFs (three 5e-tRFs and one D-tRF) were upregulated in PCa (Figure 3C–3F), and 2 tRFs (both from the 3e-tRF class) were downregulated (Figure 3G–3H).

### Specific tRF signatures can serve as prognostic marker of recurrent prostate cancer

**Figure 4:**
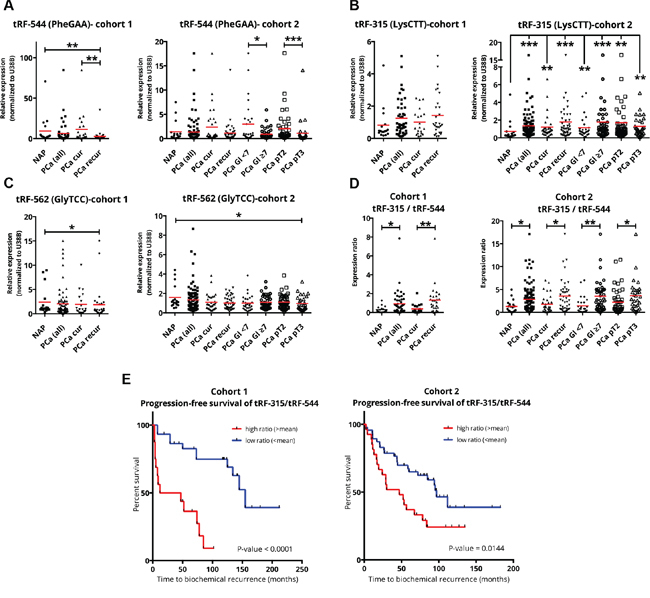
qPCR validation of tRF-544, tRF-315, and tRF-562 **A-C.** RNA expression of tRF-544 (A), tRF-315 (B), and tRF-562 (C) in cohorts of clinical samples obtained from Erasmus MC (cohort 1) and Tampere University Hospital (cohort 2). Mean values are indicated by a red line. **D.** Ratio of tRF-315 (derived from tRNA^LysCTT^) to tRF-544 (derived from tRNA^PheGAA^). **E.** Progression-free survival curves of cohort 1 and cohort 2 based on the tRF-315/tRF544 ratio. Legend: NAP, normal adjacent prostate; PCa, prostate cancer; PCa cur (PCa cured), patients with no disease recurrence after radical prostatectomy; PCa recur (PCa recurrent), patients with biochemical recurrence or metastatic progression after radical prostatectomy; Gl <7, Gleason score <7; Gl ≥7, Gleason score 7, 8 or 9; pT2, pathological stage 2; pT3, pathological stage 3; *P-value ≤0.05; **P-value ≤0.01; ***P-value ≤0.001.

The expression levels of tRFs selected for validation by qPCR were studied in a cohort of clinical samples obtained from Erasmus MC, Rotterdam (cohort 1) and a cohort of samples from Tampere University Hospital, Tampere (cohort 2). The NAP samples were identical for both cohorts and were processed independently in cohort 1 and cohort 2 to account for technical differences in sample treatment. Using custom designed primers, we could detect three tRFs (Figure 4A–4C). tRF-544 (derived from tRNA^PheGAA^) was significantly downregulated in the recurrent PCa compared to NAP or cured PCa in cohort 1 (Figure 4A). In cohort 2, tRF-544 was downregulated in PCa with Gleason score higher than 7 or in PCa with pathological stage 3 suggesting association with aggressive or late stage disease. The differential expression of this tRF was also confirmed in a second deep sequencing analysis of a sub-set of PCa samples from Tampere University Hospital (unpublished data). tRF-315 (derived from tRNA^LysCTT^) was significantly upregulated in all PCa groups of cohort 2 (Figure 4B). We could not detect statistically significant difference in the expression of tRF-315 in the smaller cohort 1. Nevertheless, there was a clear trend of tRF-315 upregulation in the PCa samples. tRF-562 (derived from tRNA^GlyTCC^) was significantly downregulated in PCa recurrent *vs*. NAP group in the cohort 1 and in the PCa pT3 *vs*. NAP group in the cohort 2 (Figure 4C).

Interestingly, tRF-544 was consistently downregulated in samples from patients that developed recurrent disease compared to samples from patients that were cured by radical prostatectomy in both cohorts. Furthermore, tRF-544 expression was lower in high- (Gleason score ≥7) compared to low-grade (Gleason score <7) tumors (Figure 4A). *Vice versa*, tRF-315 demonstrated a clear trend of upregulation in recurrent disease and its expression was higher in high-grade tumors (Figure 4B). Therefore, we reasoned that the expression of these two tRFs might be prognostic for aggressive tumor growth and disease recurrence after radical prostatectomy. We took advantage of the opposing expression patterns of these two tRFs and calculated the expression ratio tRF-315/tRF-544 for both cohorts (Figure 4D). The tRF-315/tRF-544 ratio showed significant differences, clearly distinguishing high from low grade PCa and cured from recurrent disease. Moreover, high expression ratio was significantly associated with poorer progression-free survival and shorter period to disease relapse (Figure 4E), suggesting that the tRF-315/tRF-544 ratio might represent a helpful clinical biomarker of disease progression.

## DISCUSSION

The technical progress in sequencing technologies and the rapid increase in the number of studies on sncRNA led to the discoveries of novel small RNA classes including tRFs. Since their initial identification, tRFs have been described in a plethora of species and knowledge about their function in the cell is starting to accumulate. Although several studies describe expression of tRFs in human cell lines, their actual repertoire in human tissues remains largely unknown [5, 15, 16, 22].

Here, we studied the composition and expression of tRFs in clinical PCa samples representing progressing disease stages. We found that all cytosolic tRNAs produced tRFs in the size range of 18-21 nt, representing the small class of tRFs. The longer tRNA halves were not as common, which is a consequence of the size selection (~15-35 nt) applied for the isolation of sncRNAs fraction in our study. We found a significant but weak correlation between the expression of tRFs per tRNA and the codon usage of tRNAs, suggesting that although tRF expression is dependent on the expression levels of their precursors, most likely additional mechanisms control tRF levels in the cell.

The accurate quantification of fragments derived from small RNAs in RNA sequencing data requires a precise annotation of the exact position of the fragment on its precursor transcript. To predict the locations of tRFs and quantify their expression we used the programs FlaiMapper and CLC-Bio Genomics Workbench [23]. We identified 598 unique tRFs derived from mature tRNAs. Based on the part of mature tRNA from which fragments originate, we could distinguish 5 different tRF classes. Out of these, the 5e-tRFs class was the most abundant of all and contained the highest number of unique tRFs. This finding is in agreement with other reports showing higher abundance of 5’-end derived tRFs [4, 5, 22, 32–34]. Given the role of 5’-derived tRFs in the inhibition of proteosynthesis and their role in the assembly of stress granules, a type of stress-induced cytoplasmatic foci with high concentration of untranslated mRNPs [10, 19, 30], it would be interesting to test their potential to inhibit translation and induce the assembly of stress granules *in vitro* in PCa cell lines using the set of upregulated 5’-derived tRFs identified in our study. The importance of tRFs in stress granule assembly becomes even more intriguing thanks to the latest indications that stress granules might play an important role in cancer via the negative regulation of mTORC1-hyperactivation-induced apoptosis [35]. This suggests that upregulation of tRFs might be indirectly linked with the suppression of apoptosis in cancer cells.

Our discovery cohort included patient-derived PCa samples with different clinico-pathological characteristics. The major difference in tRF expression (at least 110 unique differentially expressed tRFs) was observed between NAP and PCa tissue indicating that global upregulation of tRF production is associated with malignant transformation. Interestingly, 5e-tRFs were the predominant class upregulated in PCa. Recently, 5’-tRFs were found to induce translational inhibition in siRNA-independent way [36]. It was shown that the repressing activity of 5’-derived tRFs was dependent on the presence of a conserved “GG” dinucleotide at their 3’-end, which is a common feature of ~75% of the upregulated 5e-tRFs described in this study.

Comparing our data set with an external tRF data set of PCa cell lines generated by Lee *et al.* [5] demonstrated that all tRFs originating from 3’-pre-tRNA trailers and 32 out of 36 5’-tRFs described by Lee *et al*. were detected in our study. This suggests that tRFs in prostate (cancer) tissue and cell lines are common and discrete biological entities produced by defined molecular mechanisms. For 3’-derived tRFs we found a small overlap of only 6 out of 77 tRFs. A possible reason for that could be that 3’-derived tRFs represent a class of tRFs with a less stable expression. On the other hand, our results demonstrate that most of the downregulated tRFs are 3e-tRFs, which might be a general feature of PCa and PCa cell lines. If that is the case, the limited overlap of 3’-derived tRFs between both data sets might be caused by the less reliable detection of low expressed transcripts. Downregulation of 3’-derived tRFs might be an important event in the onset of cancer [21]. Possible functional implications of 3’-tRF down regulation in PCa can be derived from the demonstrated involvement of the 3′-derived tRF CU1276 in B-cell lymphoma cells, which suppresses proliferation and modulates the response to DNA damage [21]. Another recent study in breast cancer described several tRFs with tumor suppressor function that originate from tRNA^GlyTCC^, tRNA^GluYTC^, tRNA^AspGTC^, and tRNA^TyrGTA^ [15]. Upon induction in breast cancer cells, these tRFs suppress the stability of multiple oncogenic transcripts by sequence specific displacement of their 3′-UTRs from the RNA-binding protein YBX1. It can be assumed that downregulation of such tRFs would lead to cancer progression. Interestingly, some of the down-regulated 3’-tRFs (e.g. tRF-562 and tRF-542) in our sequencing libraries originate from the same tRNAs. Future investigations should address the functional implications on gene regulation in PCa caused by downregulation of 3’-derived tRFs and in particular tRF-544.

Due to high conservation of tRNAs we were unable to identify specific sequences that would serve as a recognition site of tRNA nucleases that discriminate and preferably cleave particular tRNAs. Recently, it was proposed that certain tRNAs switch from canonical to alternative folding and the ability to do so might cause the specific upregulation of their tRFs. For example, besides the canonical cloverleaf structure, tRNA^Ile^ has the potential to form a long hairpin [37]. tRNA^Asp^ also adopts an alternative folding in order to bind to the Alu element insertion in the 3’-UTR of the mRNA of its own aminoacyl-tRNA synthetase [38]. Since nucleotide modifications are known to affect hybridization, it is tempting to speculate to what extend they affect the alternative folding of tRNAs [39].

Finally, q-PCR analysis of tRFs differentially expressed in different grade PCa demonstrated that the expression ratio tRF-544, derived from tRNA^PheGAA^ and tRF-315 derived from tRNA^LysCTT^ effectively discriminates high from low grade prostate tumors and cured from recurrent disease. This establishes tRFs as novel candidate biomarkers for the early detection of recurrent aggressive PCa.

In conclusion, our study provides a comprehensive catalogue of tRFs expressed in various stages of PCa and provides leads for the further investigation of biological role and marker potential of these novel RNA entities in prostate cancer.

## MATERIALS AND METHODS

### Sample cohorts and processing

The discovery set used in this study consists of 10 sequencing libraries generated as previously described [24]. Briefly, each library was constructed from a total RNA pool prepared from four individual patient samples with similar pathological or genetic characteristics [40]. Different groups represent: normal adjacent prostate tissue (NAP), prostate tumors with Gleason score 6, 7, or 8 (PCa6, PCa7, PCa8), metastatic lymph nodes (LN_PCa), all obtained by radical prostatectomy; benign prostate hyperplasia tissue (BPH) obtained by cystoprostatectomy; and castration resistant prostate tumors obtained by trans-urethral resection of the prostate (TURP_PCa) [24]. The clinical parameters of each group are summarized in the Table 1. PCa groups with Gleason score 6 were divided into cured and recurrent disease groups or into groups with or without TMPRSS2-ERG fusion or ETV abnormalities. Sample material was obtained from the tissue banks of the Erasmus University Medical Center, Rotterdam, The Netherlands (Erasmus MC, Rotterdam, The Netherlands) and Tampere University Hospital (TAUH, Tampere, Finland). Collection and use of patient material was performed according to the national legislations concerning ethical requirements and approved by the Erasmus MC Medical Ethics Committee according to the Medical Research Involving Human Subjects Act (MEC-2004- 261), and the Ethical Committee of the Tampere University Hospital. Samples were snap frozen and stored in liquid nitrogen. Gleason score and the percentage of normal and cancer epithelial cells were evaluated from histological sections by two pathologists. Only samples with more than 70% of tumor cells were used for sequencing library preparation. All samples that were used for the normal prostate pool contained 0% of tumor cells. Total RNA was extracted using RNABee reagent (Campro Scientific, GmbH, Berlin, Germany) according to the manufacturer's protocol.

qPCR validation was performed in two separate cohorts. Clinical parameters are provided in Supplementary Tables 6-7). The first cohort (cohort 1) consists of 65 samples obtained from Erasmus MC. The samples were collected, handled and evaluated as mentioned in the previous paragraph. The second cohort (cohort 2) consists of 104 hormonally untreated primary prostate tumors from radical prostatectomy specimens obtained from Tampere University Hospital. The samples were confirmed to contain a minimum of 70% cancerous or hyperplastic cells by hematoxylin/eosin staining. Histological evaluation and Gleason grading for the second set were performed by a pathologist based on hematoxylin/eosin stained slides. Follow-up data was available for 74 of these samples. The use of clinical material was approved by the ethical committee of the Tampere University Hospital. Written informed consent was obtained from the subjects donating the samples. TRI-reagent (Molecular Research Center Inc., Cincinnati, OH, USA) was used to collect total RNA from the freshly frozen clinical samples, according to the manufacturer's instructions.

### RNA sequencing and expression analysis

RNA pools were outsourced for library construction and sequencing to BGI (Beijing Genomics Institute, Beijing, China). Shortly, total RNA samples were size-separated on denaturing polyacrylamide gel. RNA in the size range of 15-35 nt was recovered from the gel and used for the preparation of sequencing libraries. The libraries were sequenced by Illumina deep sequencing. The tRNA database used to map the reads was constructed from GtRNAdb: Genomic tRNA Database (http://gtrnadb.ucsc.edu/) as previously described [23, 24]. Shortly, tRNA genes with identical sequences were merged into single entries. Intronic sequences provided by GtRNAdb were removed, to allow mapping of tRFs derived from mature, spliced tRNAs. Genomic tRNAs in the database were modified by extending the 3’-ends with a single “CCA” sequence. Sequencing reads were mapped to tRNA database using CLC-Bio Genomics Workbench (Aarhus, Denmark). Subsequently, tRFs were predicted using the FlaiMapper program and our tRF database was constructed [23]. The final read counts used for expression analysis were generated by mapping the sequencing reads to the tRF database. tRFs derived from 5’-pre-tRNA leaders (5’U-tRFs) and 3’-pre-tRNA trailers (3’U-tRFs) were identified by mapping the sequencing reads to a tRNA reference database in which the genomic sequence of each tRNA gene was extended by 50 bp on both sides. The length, position and type of tRF were calculated from the sum of the read counts of the following groups: NAP, PCa6_cur, PCa6_nofusion, PCa6_TERG, PCa6_recur, PCa7_recur, PCa8_recur, TURP_PCa, and LN_PCa. To identify differentially expressed tRFs, read counts were normalized as “parts per million” and Kal's Z-test on proportions followed by Bonferroni correction was subsequently performed. The generated adjusted p-values lower than 0.05 were considered significant.

### Quantitative real-time PCR and statistics

Total RNA extracted from clinical samples was reverse transcribed using miRCURY Universal cDNA Synthesis kit (Exiqon, Vedbaek, Denmark), by adding a poly-A tail to the RNA template and then reverse transcribing the template to cDNA using poly-T primer with a 3’ degenerate anchor and a 5’ universal tag. The provided UniSp6 spike-in RNA was added to the reverse transcription reaction to control for the efficiency of the reaction. Template amplification was performed using miRCURY LNA™ SYBR^®^ Green Master Mix (Exiqon, Vedbaek, Denmark). Briefly, the cDNA template is amplified using tRF-specific custom LNA™ primers (Exiqon, Vedbaek, Denmark) with a forward primer spanning a large 5’ portion of the template and a reverse primer spanning the 5’ universal tag, the poly-T stretch and several templated nucleotides from the 3’end of the template. Primer pairs were designed to be specific for the tRF. Primer specificity was monitored by performing melting curve analysis and comparing its profile and obtained melting temperature with the melting temperature indicated for each amplicon. The names of tRFs, their sequences and custom primer set design IDs (Exiqon Vedbaek, Denmark) are given in Table 2. Quantitative real-time PCR (qPCR) was performed on an Applied Biosystems ABI 7900 thermocycler (Applied Biosystems, Waltham, Massachusetts, USA) for the cohort 1 and on Bio-Rad CFX96 Real Time System (Bio-Rad Laboratories, Hercules, California, USA) for the cohort 2. Data were analyzed using the ΔΔCT method and the expression of each tRF was normalized against the small nucleolar RNA SNORD38B (Reference gene primer set 2039, Exiqon, Vedbaek, Denmark). Statistical significance of qPCR expression data was assessed using Mann-Whitney U test. The log-rank test was used to compare progression-free survival distributions of the tumor samples. P-values lower than 0.05 were considered statistically significant. Statistical analysis was performed using GraphPad Prism version 6.0g for Mac OS X (GraphPad Software, La Jolla California USA, www.graphpad.com”).

**Table 2:** tRFs selected for validation by qPCR

tRF ID	tRNA isotype	Anticodon	Sequence 5’- 3’	Custom primer set Design ID
tRF-544	Phe	GAA	TCCCTGGTTCGATCCCGGGTTTCGGCA	205919-1
tRF-159	Arg	CCT	ATGGATAAGGCATTGGCCT	205895-1
tRF-368	Arg	TCT	GGCTCCGTGGCGCAATGGA	205891-1
tRF-562	Gly	TCC	TCGATTCCCGGCCAACGC	205924-1
tRF-542	Glu	CTC	TCCCTGGTGGTCTAGTGGTTAG	205899-1
tRF-315	Lys	CTT	CCCGGCTAGCTCAGTCGGTAGAGCATGG	36855-1

## SUPPLEMENTARY FIGURES AND TABLES




