# Supplementary material for: A comprehensive repertoire of tRNA-derived fragments in prostate cancer
Source: Oncotarget. 2016 Mar 23;7(17):24766–77. doi: 10.18632/oncotarget.8293 (PMC5029740; doi:10.18632/oncotarget.8293)
Supplement: Supplementary file 1 [file oncotarget-07-24766-s001.pdf]

SUPPLEMENTARY FIGURES AND TABLES

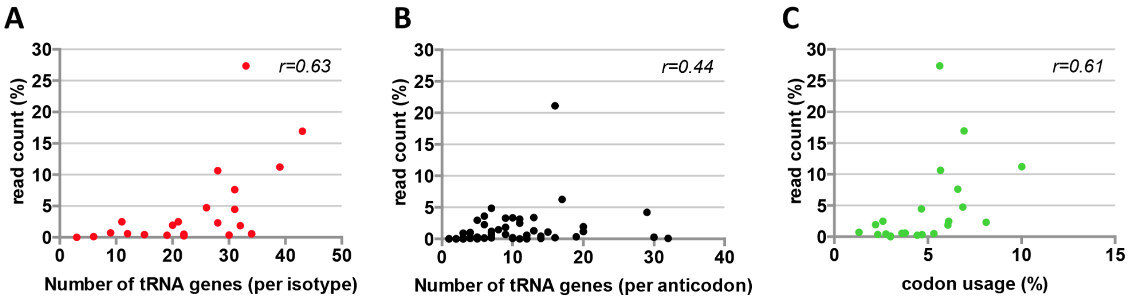

Supplementary Figure S1: Correlation of tRF levels with their estimated precursor levels. Scatter plots depicting the relationship between the expression of tRFs and their precursors. Correlation between the percentage of tRF read counts and the number of tRNA genes per isotype **A.** anticodon **B.** or codon usage **C.** Spearman correlation coefficient (r) is indicated in the graph. All correlations were significant (P-value <0.05). Due to the high sequence similarities between tRNAs, several reads mapped to multiple tRNA loci and thus were omitted from the analysis (percentage of tRFs mapped to multiple loci in figure: A=2%, B=21%, C=2%).

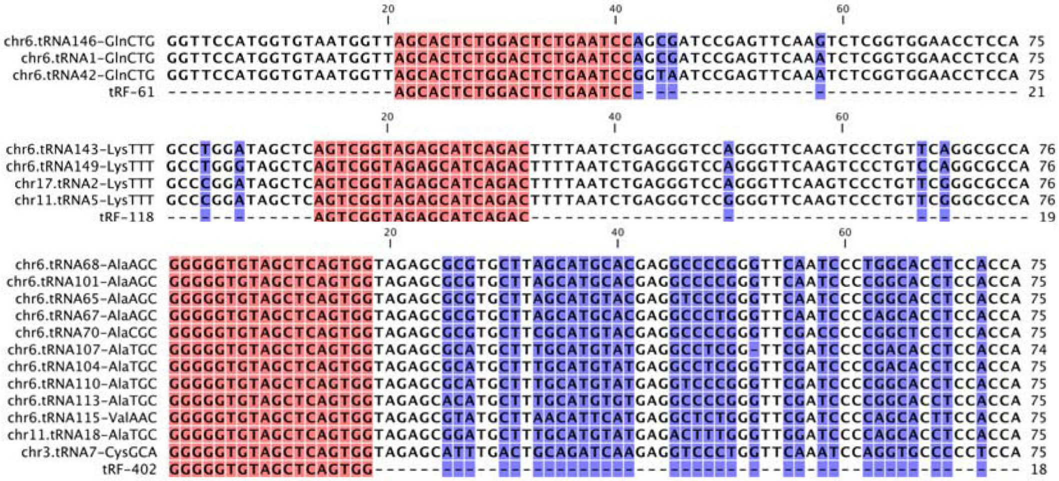

Supplementary Figure S2: Sequence alignments of three tRF examples (tRF-61, tRF-118, and tRF-402) that mapped to different tRNA loci. The tRF location is highlighted in red. Nucleotide positions that are dissimilar between tRNA sequences are highlighted in blue.

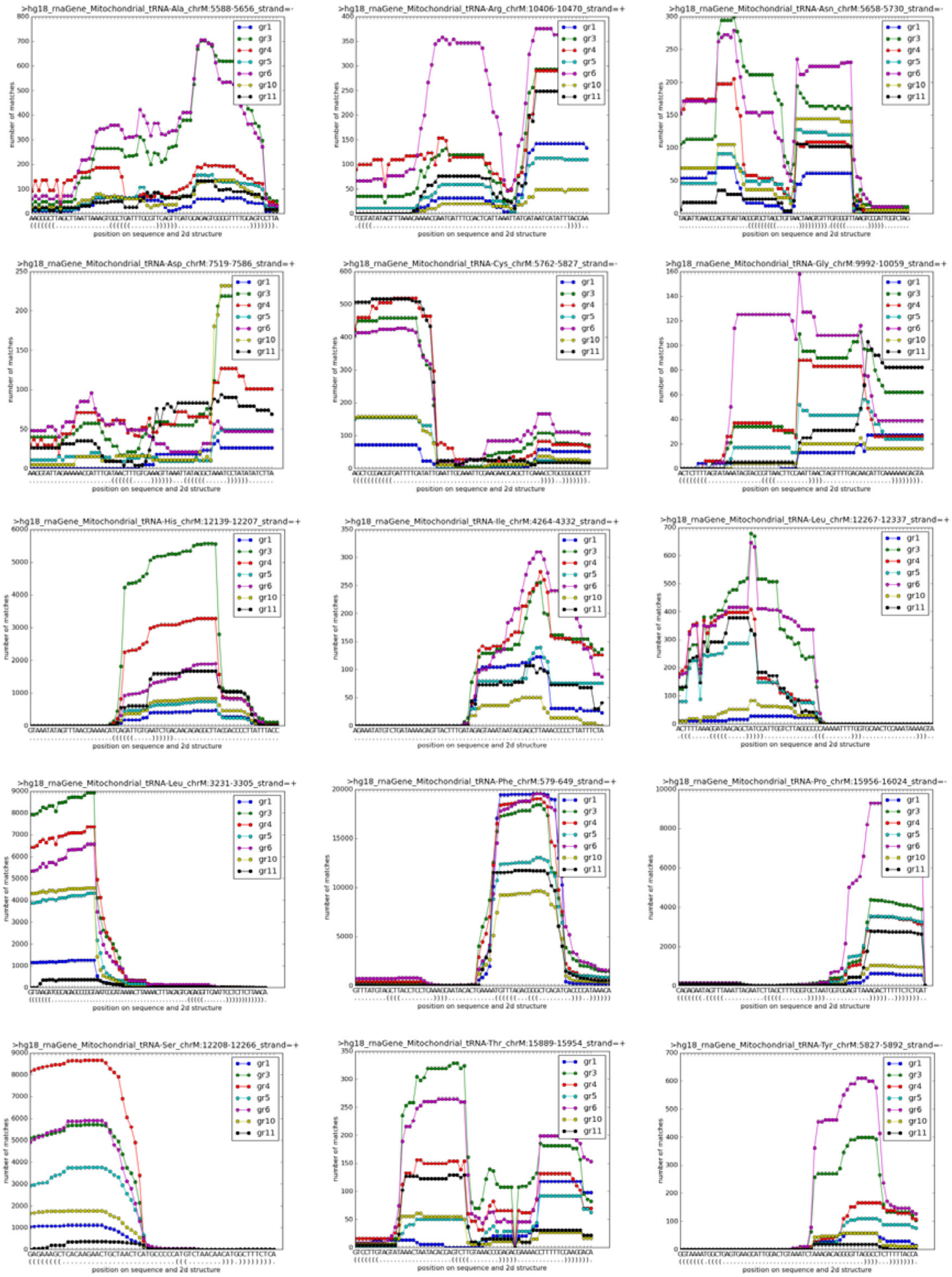

Supplementary Figure S3: Fragments derived from mitochondrial tRNAs. Graphs showing the coverage of tRF nucleotides projected on mature mtRNA for each study group, reveal the complicated fragmentation pattern of mitochondrial tRNAs. The x-axis shows the sequence of the mtRNA (in the 5′ to 3′ direction) to which tRFs were mapped. The experimental groups are shown with different colors. Legend: gr1-NAP; gr3-PCa6_cur; gr4-PCa6_recur; gr5-PCa7_recur; gr6-PCa8_recur; gr10-PCa6_nofusion; gr11-PCa6_TERG.

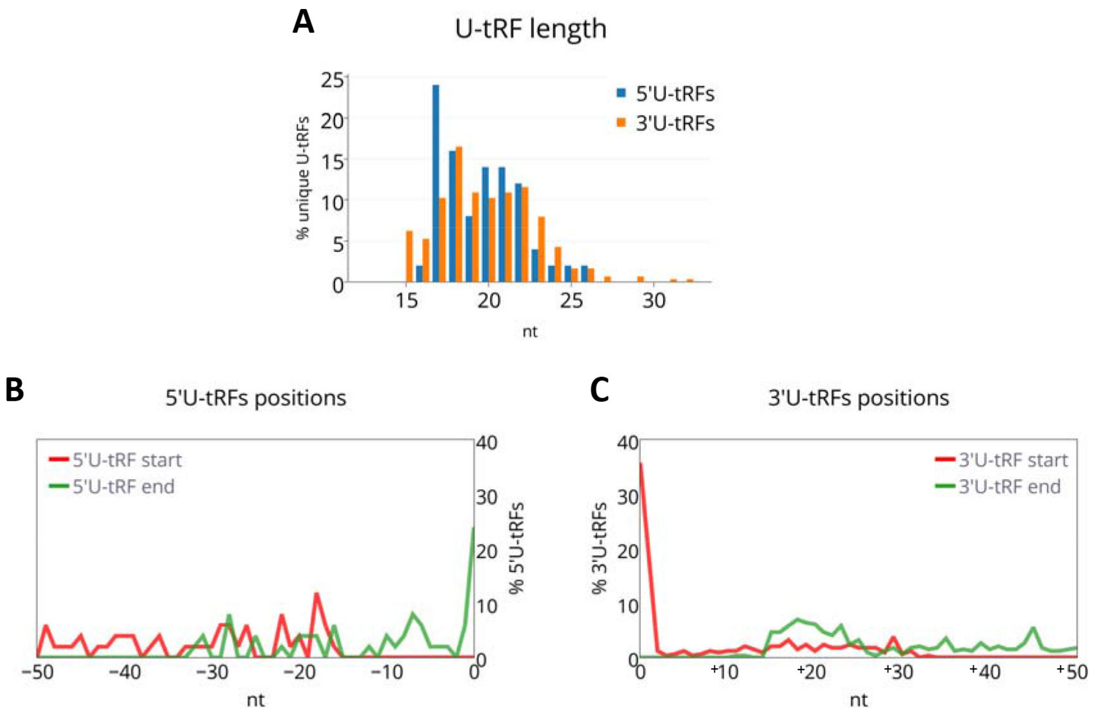

Supplementary Figure S4: U-tRF types in prostate cancer. The size distribution and location of 5’-pre-tRNA leaders (5’U-tRFs) and 3’-pre-tRNA trailers (3’U-tRFs) was analyzed. **A.** U-tRF length as based on the uniqueness. **B-C.** Start (red line) and end (green line) positions of 5’U-tRFs (B) and 3’U-tRFs (C) on the 5’-leaders and 3’-trailers of pre-tRNAs. The values on X-axis represent number of nucleotides from the start or end of mature tRNA sequence.

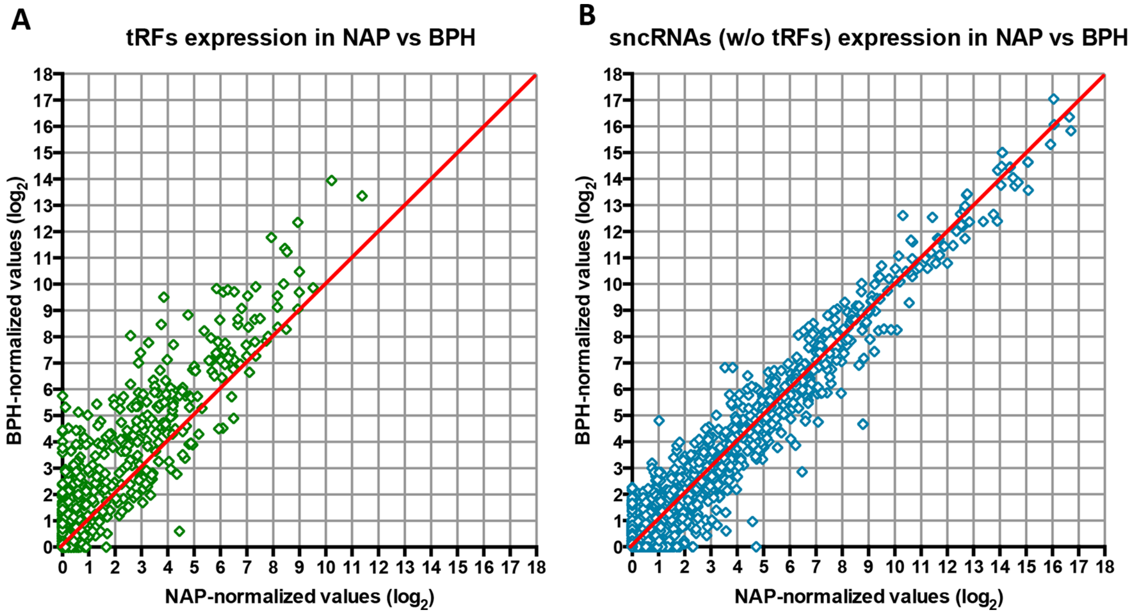

Supplementary Figure S5: tRF expression is affected in BPH. Scatter plots of log2-transformed normalized values of tRFs **A.** and other sncRNAs **B.** including miRNAs, snoRNA and sdRNA, in NAP *vs* BPH group. The red line indicates no change in the expression between NAP and BPH.

Supplementary Table S1: A list of tRFs identified across our dataset. tRF ID number, sequence, genomic locus (loci) that they mapped to, their position on the locus and the locus sequence are indicated

See Supplementary File: 1

Supplementary Table S2: A list of tRFs derived from 3'-pre-tRNA trailers and 5'-pre-tRNA leaders

See Supplementary File: 1

Supplementary Table S3: Read count values of each tRF in individual study group

See Supplementary File: 1

Supplementary Table S4: Differentialy expressed tRFs and their adjusted p-values

See Supplementary File: 1

Supplementary Table S5: A list of recurrently changed tRFs across PCa6_recur, PCa7_recur and PCa8_recur groups

See Supplementary File: 1

Supplementary Table S6: Clinical parameters of samples in the cohort 1

See Supplementary File: 1

Supplementary Table S7: Clinical parameters of samples in the cohort 2

See Supplementary File: 1
